# Excess mortality and the COVID-19 pandemic: causes of death and social inequalities

**DOI:** 10.1186/s12889-022-14785-3

**Published:** 2022-12-07

**Authors:** Jieun Oh, Jieun Min, Cinoo Kang, Ejin Kim, Jung Pyo Lee, Ho Kim, Whanhee Lee

**Affiliations:** 1 Department of Public Health Science, Graduate School of Public Health, Seoul National University, Seoul, 08826 Republic of Korea; 2 Department of Environmental Medicine, College of Medicine, Ewha Womans University, Seoul, Republic of Korea; 3 Graduate Program in System Health Science and Engineering, College of Medicine, Ewha Womans University, Seoul, Republic of Korea; 4 Institute of Health and Environment and Graduate School of Public Health, Seoul National University, Seoul, Republic of Korea; 5 Department of Internal Medicine, Seoul National University Boramae Medical Center, Seoul, Republic of Korea; 6 School of Biomedical Convergence Engineering, College of Information and Biomedical Engineering, Pusan National University, Yangsan, 50612 Republic of Korea

**Keywords:** Excess mortality, COVID-19, Cause-specific mortality, Social inequality

## Abstract

**Background:**

During the coronavirus diseases 2019 (COVID-19) pandemic, population’s mortality has been affected not only by the risk of infection itself, but also through deferred care for other causes and changes in lifestyle. This study aims to investigate excess mortality by cause of death and socio-demographic context during the COVID-19 pandemic in South Korea.

**Methods:**

Mortality data within the period 2015–2020 were obtained from Statistics Korea, and deaths from COVID-19 were excluded. We estimated 2020 daily excess deaths for all causes, the eight leading causes of death, and according to individual characteristics, using a two-stage interrupted time series design accounting for temporal trends and variations in other risk factors.

**Results:**

During the pandemic period (February 18 to December 31, 2020), an estimated 663 (95% empirical confidence interval [eCI]: -2356–3584) excess deaths occurred in South Korea. Mortality related to respiratory diseases decreased by 4371 (3452–5480), whereas deaths due to metabolic diseases and ill-defined causes increased by 808 (456–1080) and 2756 (2021–3378), respectively. The increase in all-cause deaths was prominent in those aged 65–79 years (941, 88–1795), with an elementary school education or below (1757, 371–3030), or who were single (785, 384–1174), while a decrease in deaths was pronounced in those with a college-level or higher educational attainment (1471, 589–2328).

**Conclusion:**

No evidence of a substantial increase in all-cause mortality was found during the 2020 pandemic period in South Korea, as a result of a large decrease in deaths related to respiratory diseases that offset increased mortality from metabolic disease and diseases of ill-defined cause. The COVID-19 pandemic has disproportionately affected those of lower socioeconomic status and has exacerbated inequalities in mortality.

**Supplementary Information:**

The online version contains supplementary material available at 10.1186/s12889-022-14785-3.

## Background

The coronavirus disease 2019 (COVID-19) pandemic has posed a serious and persistent threat to global public health and has brought unprecedented changes to daily life. Moreover, the unprecedented scope of the worldwide pandemic has led to extraordinary demands on the healthcare system, resulting in critical shortages of medical resources and serious reductions in social capital [1]. Thus, to alleviate the burden of the pandemic, numerous countries have implemented a number of non-pharmaceutical interventions, such as social distancing and individual hygiene practices, although there have been differences in both the intensity and effectiveness of these interventions [2].

Along with the risk of infection itself, the collateral effects of the pandemic have affected population health and may be associated with mortality risk through various pathways [3–10]. During the pandemic, medical resources and mobilisation have been concentrated on patients with confirmed COVID-19, and less critical medical services for non-COVID-19 patients with less severe or less urgent diseases and/or those at a lower age-related risk have frequently been postponed or cancelled [3, 4]. In addition, previous studies have reported that medical accessibility is closely associated with socioeconomic status [5, 6], and that changes in lifestyle and health behaviours during the pandemic (such as wearing masks and engaging in fewer social and physical activities) might exhibit non-uniform effects on people with heterogeneous characteristics, with differential findings according to disease type, age, sex, educational level, and marital status [5, 7–10]. In sum, these results indicate that limited access to medical services during the pandemic might disproportionally affect individuals depending on their medical and socioeconomic status.

Excess mortality, defined as the increase in deaths compared to the expected number of deaths, has been widely used as a representative indicator for the damage caused by the pandemic with respect to human health [11]. Multiple studies have reported excess mortality attributed to the pandemic [12–16]. Nevertheless, although it can be strongly conjectured that the health damage related to the pandemic is heterogeneous among populations, most previous studies on pandemic-associated excess mortality have solely addressed total mortality (i.e., without consideration of causes of death and variation according to individual characteristics), and only a few studies have evaluated cause-, sex-, age-, race-, or income level-specific impacts [13–18]. However, an in-depth examination of cause-specific and individual-specific excess mortality can provide scientific evidence informing interventions in vulnerable populations as well as public health resource allocation. We note that South Korea (hereafter termed Korea) has been evaluated as a country that has successfully responded to the pandemic with widespread testing and epidemiological investigations at the initial pandemic stage; therefore, assessing excess deaths occurring due to the pandemic in Korea can provide an informative evidence base for public health researchers and policymakers [19, 20]. Nevertheless, although the socio-demographic characteristics may be involved in shaping the consequences of COVID-19, they have not been considered in previous studies in Korea. Hence, this study aimed to investigate nationwide excess mortality during the 2020 pandemic period in Korea and to identify relevant factors that could affect excess mortality, including causes of death and individual characteristics (i.e., age, sex, educational level, and marital status). We hypothesised that we would observe social inequities in mortality outcomes during the pandemic period.

## Methods

### Statement on guidelines

This study complies with relevant guidelines and regulations. All our dataset has been publicly available and did not include any identifiable information. This study was carried out using only data from Statistics Korea, Korea Disease Control and Prevention Agency, and the Korea Meteorological Administration, and there was no direct involvement of participants. Thus, patient consent procedures and ethics approval were not required for this study.

### Data

We downloaded data on deaths occurring between 2015–2020 in all 16 regions of Korea from Statistics Korea [21]; the information available for death case with individual characteristics: date of death, age, sex, education level, marital status, and underlying causes of death (classified according to the 10^th^ Revision of the International Classification of Diseases; ICD-10). From this data, we calculated the daily number of deaths from all causes and by eight leading causes of death and the individual characteristics. We also collected data on confirmed cases of COVID-19 occurring in 2020 from Korea Disease Control and Prevention Agency [22]. Data on daily average temperatures in 2015–2020 across 16 regions in Korea were obtained from the Korea Meteorological Administration [23].

### Causes of death

We considered deaths from all causes as well as due to eight leading causes of death based on the main category (i.e. the first letter of the code) of the ICD-10 code, including infectious diseases, neoplasms, metabolic diseases, circulatory diseases, respiratory diseases, genitourinary diseases, ill-defined causes, and external causes (see Supplementary Table S1 for more detailed information). The ICD-10 codes for COVID-19 deaths (U07.1, U07.2) were excluded from this study to identify the collateral impacts of the pandemic on mortality and the COVID-19 deaths accounted for only a small portion of total deaths (950 in total; 0.3% of total deaths) (see Supplementary Table S2 for more detailed information).

### Individual characteristics

To investigate the impact of COVID-19 on excess deaths according to socio-demographic factors, death cases were aggregated by sex, age (< 65, 65–79, and ≥ 80 years), education level (elementary school, middle school, high school, and ≥ college), and marital status (single, married, other [e.g., divorced, widowed]).

### Two-stage analyses

We conducted two-stage interrupted time-series analyses to quantify the excess risk of mortality during the COVID-19 pandemic period as compared with the pre-pandemic period in Korea, following a methodological approach delineated in previous studies [24, 25].

In the first stage, a quasi-Poisson regression model was applied to each of the 16 regions in Korea [26]. In the time-series analysis, the usage of other methods (e.g., autoregressive integrated moving average model) [27] was limited, because we used the death count data which takes values in non-negative integers. Thus, we performed quasi-Poisson regression with seasonality and long-term trend adjustments using a spline function [26]. We used the number of days from the first COVID-19 confirmed case to estimate the time-varying risk during the outbreak period (January 20 to December 31, 2020). We included a linear term for date to model long-term trends, a term for days of the year to control for seasonality, and dummy indicators for the day of the week to adjust for variation by week. We also modelled the relationship between average daily temperature readings and mortality using a distributed lag nonlinear model [28, 29]. The characteristics of the 16 regions considered in this study are presented in Supplementary Table S2.

In the second stage, we pooled the region-specific coefficients of excess risk obtained during the COVID-19 period to the nationwide level using a mixed-effects multivariate meta-analysis approach [30]. The best linear unbiased prediction (BLUP) was then calculated for each of the 16 regions to stabilise the variability due to the large differences in population size between regions, leading to more precise estimates [31].

More detailed information on the two-stage interrupted time-series design employed herein can be found in the Supplementary Material.

### Quantification of excess deaths

The relative risk (RR) of excess mortality was calculated to quantify excess deaths attributable to COVID-19. We obtained the predicted values for excess mortality via BLUP region-specific estimates and exponentiated these values to obtain the RR for each day of the outbreak period in each region. The daily number of excess deaths was computed as $n*(RR-1)/RR$, where $n$ represents the number of deaths per day. We aggregated the daily excess number of deaths by pandemic wave and plateau for each of the 16 regions and for the entirety of Korea. The definition of the COVID-19 period is presented in the Supplementary Material. We computed empirical 95% confidence intervals (eCIs) for the coefficients using Monte Carlo simulations.

We repeated the main analysis described earlier for stratified analyses to estimate the number of excess deaths for each eight leading causes of death and individual characteristics.

### Sensitivity analyses

We conducted several sensitivity analyses to assess the robustness of our findings. More specifically, we applied five and six internal knots in the quadratic B-spline function for days since the first COVID-19 confirmed case, four and six knots in the cyclic B-spline function for days of the year, and 14 and 28 days of lag period in the distributed lag nonlinear model.

## Results

### Excess all-cause mortality

Total deaths and estimated excess deaths during the pandemic period between February 18 and December 31, 2020 are reported in Table 1. During this period, 260,432 deaths were reported in Korea. The number of excess deaths from all causes was estimated as 663 (95% eCI: -2356–3584), indicating that there was no evident excess in total mortality during the pandemic period as compared with the pre-pandemic period.

**Table 1:** Number of total deaths, estimated excess deaths and percentage excess in mortality (with 95% empirical confidence intervals) during the COVID-19 pandemic period (February 18 to December 31, 2020) in Korea

		Total deaths	Excess deaths	Percentage excess
Total		260,432	663 (-2356 to 3584)	0.3 (-0.9 to 1.4)
Sex	Males	141,565	531 (-975 to 2018)	0.4 (-0.7 to 1.4)
	Females	118,867	175 (-1873 to 2128)	0.1 (-1.6 to 1.8)
Age	< 65	57,835	-232 (-1179 to 631)	-0.4 (-2 to 1.1)
	65–79	76,429	941 (88 to 1795)	1.2 (0.1 to 2.4)
	≥ 80	126,168	-748 (-2894 to 1445)	-0.6 (-2.2 to 1.2)
Education	≤ Elementary school	121,571	1757 (371 to 3030)	1.5 (0.3 to 2.6)
	Middle school	34,174	407 (-539 to 1278)	1.2 (-1.6 to 3.9)
	High school	55,868	-825 (-1812 to 176)	-1.5 (-3.1 to 0.3)
	≥ College	33,989	-1471 (-2328 to -589)	-4.1 (-6.4 to -1.7)
Marital status	Single	20,984	785 (384 to 1174)	3.9 (1.9 to 5.9)
	Married	118,219	354 (-709 to 1332)	0.3 (-0.6 to 1.1)
	Others (divorced/widowed)	120,788	-415 (-2759 to 1679)	-0.3 (-2.2 to 1.4)
Cause of death	Certain infectious and parasitic diseases	8132	222 (-797 to 1127)	2.8 (-8.9 to 16.1)
	Neoplasms	72,568	33 (-1821 to 2028)	0.0 (-2.4 to 2.9)
	Endocrine, nutritional and metabolic diseases	8577	808 (456 to 1080)	10.4 (5.6 to 14.4)
	Diseases of circulatory system	53,325	744 (-1021 to 2417)	1.4 (-1.9 to 4.7)
	Diseases of respiratory system	29,815	-4371 (-5480 to -3452)	-12.8 (-15.5 to -10.4)
	Diseases of the genitourinary system	7956	81 (-872 to 920)	1.0 (-9.9 to 13.1)
	Symptoms, signs and abnormal clinical and laboratory findings, not elsewhere classified	27,548	2756 (2021 to 3378)	11.1 (7.9 to 14)
	Injury, poisoning and certain other consequences of external causes	23,024	-423 (-1578 to 641)	-1.8 (-6.4 to 2.9)

### Excess cause-specific mortality

Nevertheless, we found heterogeneous excess deaths when evaluating cause-specific deaths (Table 1). For example, the number of deaths related to respiratory diseases decreased by 4371 due to the pandemic (95% eCI: 3452–5480), corresponding to a 12.8% percentage decrease in this mortality outcome (10.4%–15.5%). However, excess deaths due to metabolic diseases and “ill-defined cause” diseases attributable to the pandemic increased by 808 (456–1080) and 2756 (2021 to 3378), corresponding to percentage increases of 10.4% (5.6%–14.4%) and 11.1% (7.9%–14%), respectively.

### Excess mortality by individual characteristics

**Fig. 1:**
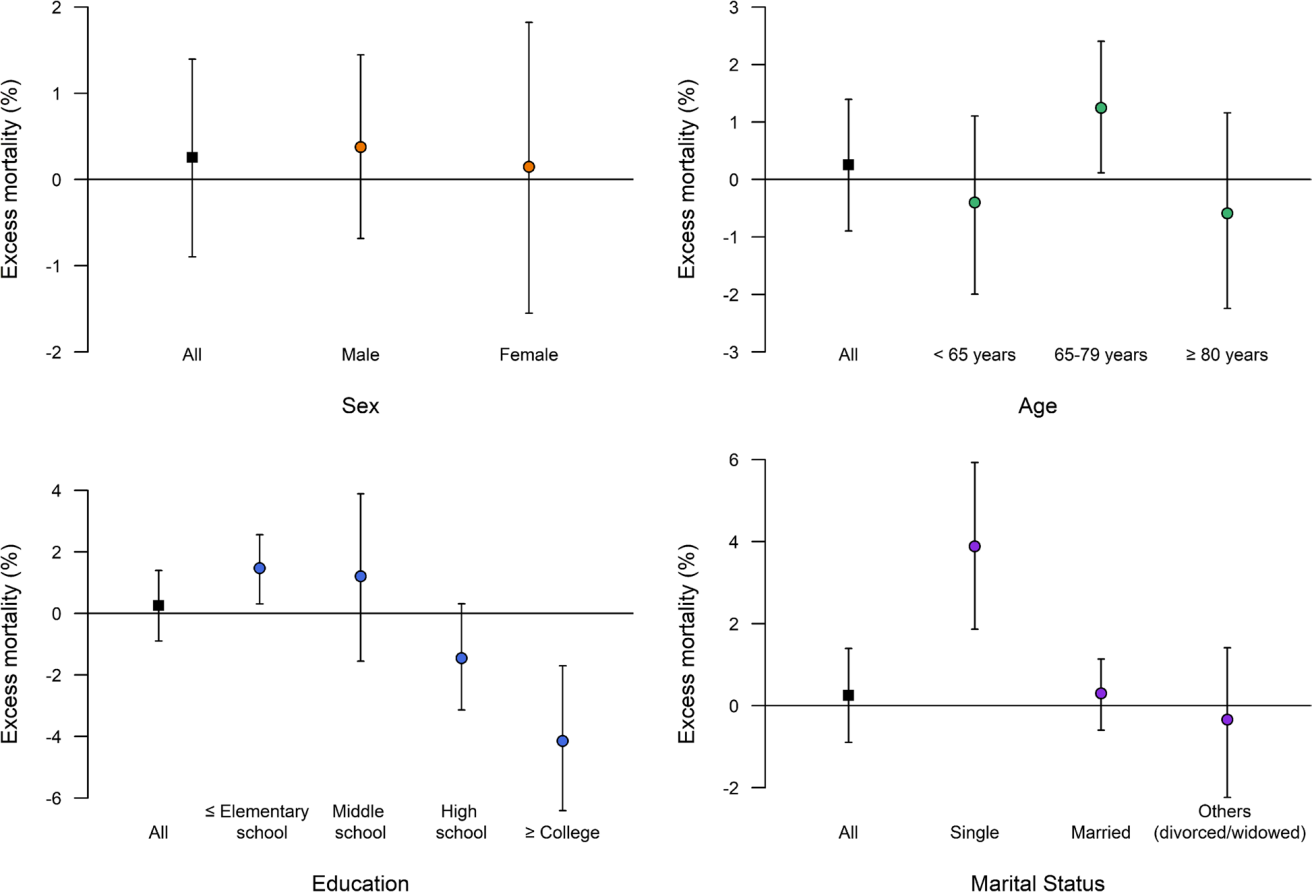
Percentage excess in mortality (with 95% empirical confidence interval) during the COVID-19 pandemic period (February 18 to December 31, 2020) in Korea by sex, age, education, and marital status

We found that the impact of the pandemic on mortality was disproportionate according to socio-demographic characteristics (Table 1, Fig. 1). For example, the excess mortality attributable to the pandemic was prominent in those aged 65 to 79 years (excess deaths 941, 95% eCI: 88–1795; percentage excess 1.2%, 95% eCI: 0.1%–2.4%), those with an elementary school or lower educational level (1757, 371–3,030; 1.5%, 0.3%–2.6%), and in the single population (785, 384–1174; 3.9%, 1.9%–5.9%). However, we found a decrease in the mortality rate during the pandemic in people with a college-level or higher educational attainment (1471, 589–2328; 4.1%, 1.7%–6.4%).

### Temporal trends in excess mortality

**Fig. 2:**
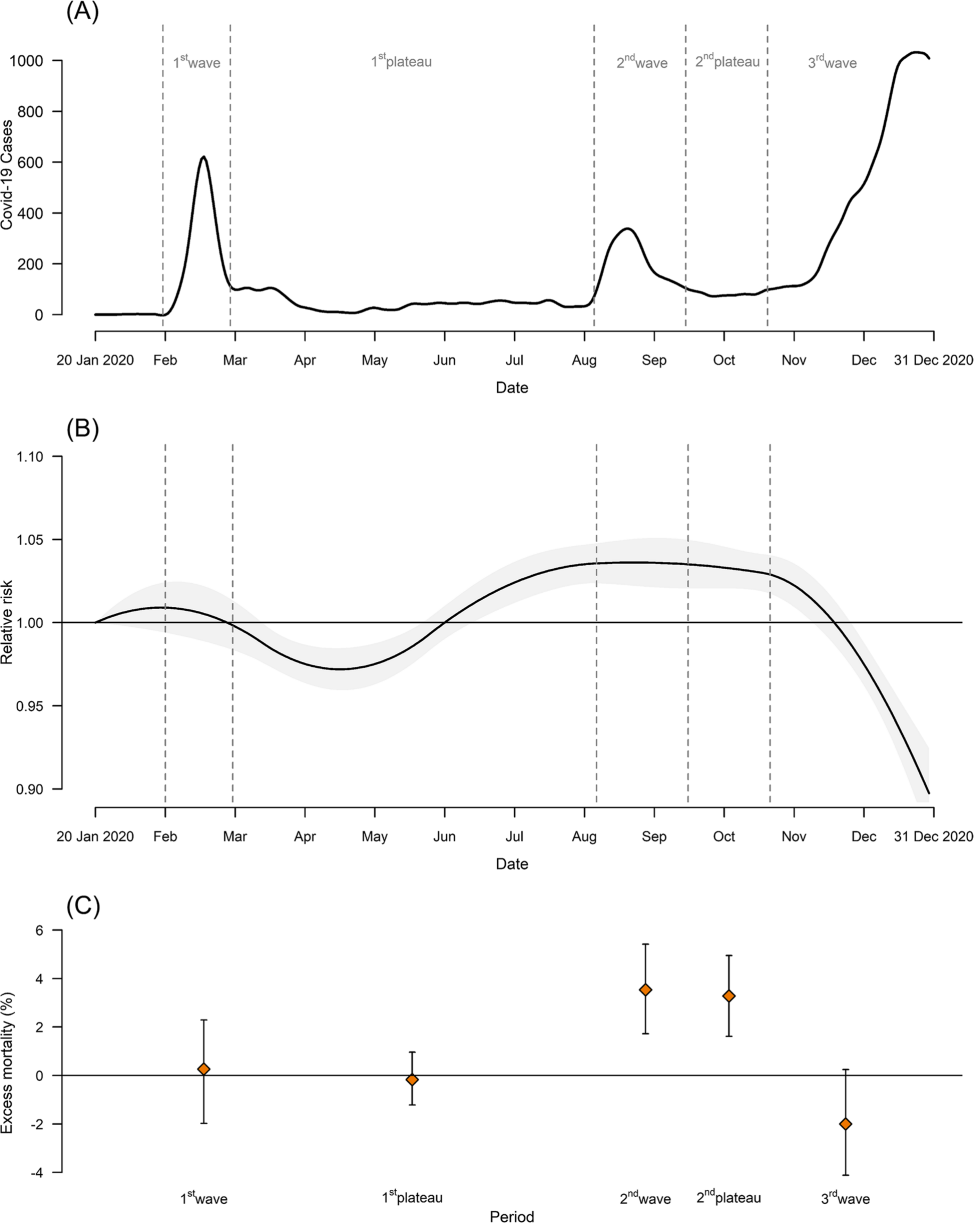
Temporal trends in 7-day moving average of COVID-19 confirmed cases (**a**), excess risk (relative risk, RR) with a band corresponding to the 95% empirical confidence interval (**b**), and percent excess in mortality by phases of the pandemic (**c**) during the period January 20 to December 31, 2020, in Korea

For all-cause deaths, we found fluctuations and inconsistent patterns in the temporal trend for excess risk (RR) and the percent excess in mortality across waves and plateaus of the COVID-19 pandemic (Fig. 2). The excess risk of mortality started decreasing from the beginning of the 1^st^ plateau, then gradually increased until it reached its peak in the 2^nd^ wave. Subsequently, the risk continued to decrease, with a sharp decline evident during the 3^rd^ wave. For cause-specific deaths, three types of deaths showed obvious and consistent patterns during the pandemic period. Namely, we found a decrease in mortality related to respiratory diseases and an increase in mortality due to both metabolic diseases and diseases with ill-defined causes (see Supplementary Table S3).

### Excess mortality both by causes of death and individual characteristics

**Fig. 3:**
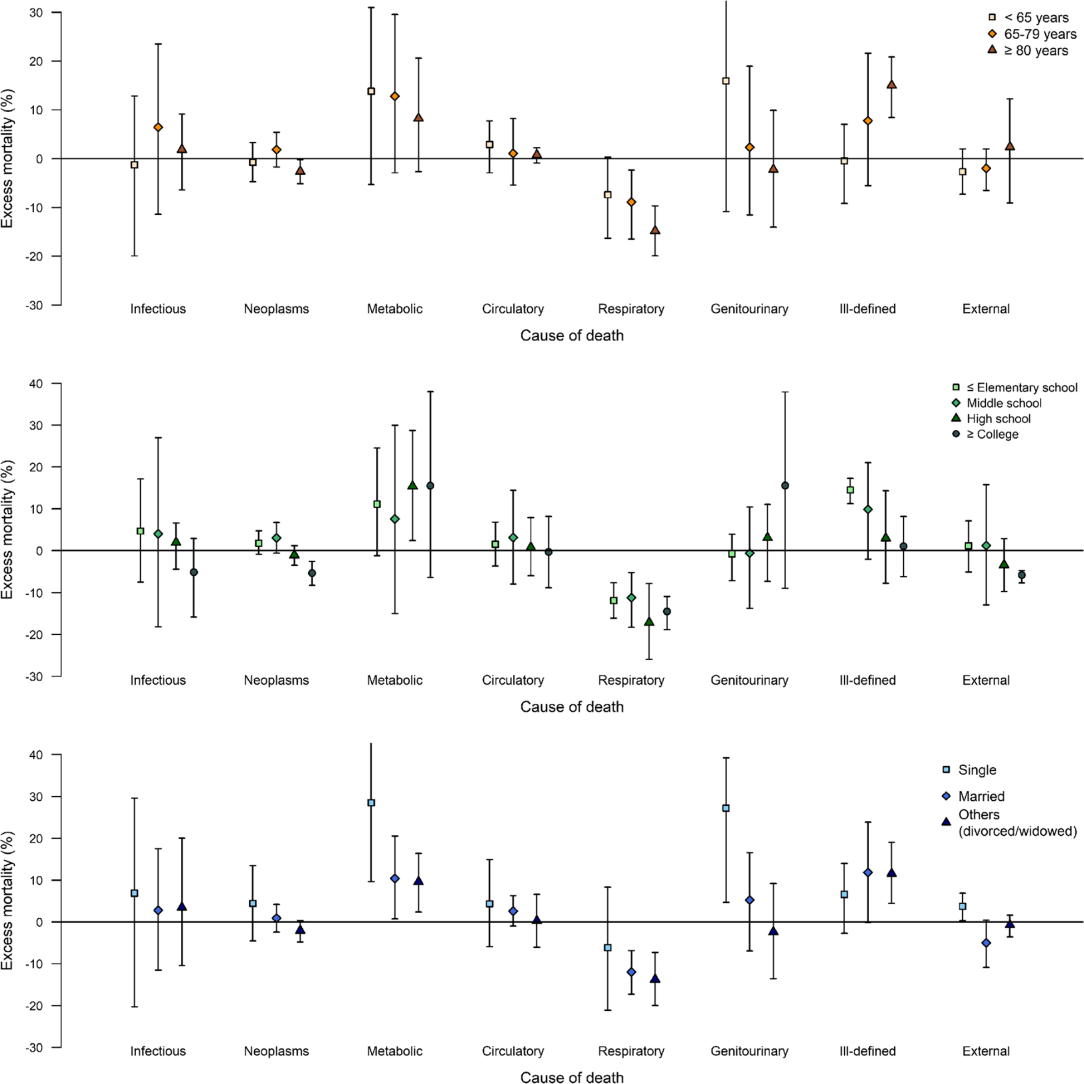
Percentage excess in mortality (with 95% empirical confidence interval) during the COVID-19 pandemic period (February 18 to December 31, 2020) in Korea for each cause of death by age, education, and marital status. Abbreviations: Infectious = Certain infectious and parasitic diseases, Metabolic = Endocrine, nutritional and metabolic diseases, Circulatory = Diseases of circulatory system, Respiratory = Diseases of respiratory system, Genitourinary = Diseases of genitourinary system, Ill-defined = Symptoms, signs and abnormal clinical and laboratory findings, not elsewhere classified, External = Injury, poisoning and certain other consequences of external cause

Excess mortality due to cause-specific deaths and according to individual characteristics is shown in Fig. 3 and Supplementary Table S4. Respiratory disease-related mortality showed an evident reduction during the pandemic in all age groups. However, excess mortality due to ill-defined causes prominently increased in those aged 80 years or older with percentage excess of 15% (8.4%–20.8%). Moreover, across all specific causes, an increase in mortality due to the pandemic was generally more evident in those with lower education levels (high school or lower), while a decrease in mortality was more obvious in those with higher education levels (college or higher). The exception to this trend was with regard to respiratory disease deaths, which showed reduced mortality across all educational groups. This pattern (i.e., a higher excess mortality in those with lower education levels) was more prominent for metabolic and ill-defined causes of death. Excess mortality attributable to the pandemic was generally more pronounced across all specific causes in the single population.

### Sensitivity analysis results

Sensitivity analyses were performed to assess whether these findings were consistent with the modelling specifications; the sensitivity analysis results revealed the robustness of our main results (see Supplementary Tables S5 and S6).

## Discussion

This study investigated nationwide excess mortality during the COVID-19 pandemic in Korea according to cause of death and individual characteristics. In the total population, although there were no substantial excess deaths evident during the pandemic period when estimated except for deaths from COVID-19, we found disproportionate impacts of the pandemic on mortality by cause of death, education level, and marital status. In general, the excess mortality attributable to the pandemic was more evident in deaths from metabolic and ill-defined diseases, in those with lower education levels, and in the single population.

To our knowledge, several previous studies have evaluated trends in excess mortality during the first year of the pandemic. For example, a study evaluating mortality trends in 29 industrialised countries reported an increase in mortality due to the pandemic [13]. Another study evaluating trends in 67 countries also showed that most countries experienced an increase in mortality during the pandemic, with the exception of some countries with higher testing capacities [32]. Nevertheless, we did not detect evident increases in mortality due to the pandemic in Korea in the current study. We conjecture that this pattern may be closely associated with the early and extensive testing and comprehensive epidemiological investigations implemented in Korea in response to the pandemic, which have been identified as effective countermeasures in reducing the spread and mortality rate associated with COVID-19 [19, 20].

Although some previous studies have reported excess mortality during the pandemic in Korea, the results have been mixed. For example, some studies showed no evident increase in annual deaths in 2020 [13, 17], whereas another study reported a decrease in mortality in 2020 [32]. However, these previous studies were based on weekly or monthly mortality data. Thus, we believe that our study, which was based on daily data and employed a cutting-edge standardised time-series analysis, can provide more precise estimates than these prior investigations.

This study identified that the impact of the pandemic on mortality was disproportionate in accordance with cause of death, age group, educational level, and marital status. First, we found that a large decrease in mortality from respiratory diseases during the pandemic was the major factor in the non-increased mortality pattern evident in this study, and that this trend may have offset increases in the mortality rate due to metabolic and ill-defined diseases that occurred during the pandemic period. This result is consistent with that of a previous study that examined the decline in the incidence and mortality of respiratory diseases in Korea during the pandemic period [8, 17]. We note that, the Korean government has generally implemented high levels of social distancing, personal hygiene, and mask-wearing since the initial stage of the pandemic, and that these are major factors in the decrease in respiratory virus infection evident in this country [19, 20].

However, we found that prominent excess mortality attributable to the pandemic was observed in metabolic disease-related deaths, and that this excess mortality was more evident in those with lower education levels and single marital status. These results could partly be explained by the unintended impact of interventions against the pandemic [7]. For example, social distancing and fewer outdoor activities could increase time spent indoors and lead to worsened health behaviours, such as unhealthier diets and less exercise. Moreover, restricted and reduced accessibility to medical services during the pandemic could negatively affect consistent care for patients with chronic metabolic diseases and the impacts of this decreased accessibility to medical services could be more pronounced in those with low socioeconomic status, which may result in fewer hospital visits and medications.

In addition, we found that excess mortality due to the pandemic was evident in regard to deaths from ill-defined causes. Interestingly, we found that the number of deaths due to ill-defined causes increased throughout 2020 in Korea, and that this pattern was not observed for other causes of death. Moreover, this increasing pattern was more pronounced in those aged 80 years or older. From our study data, we found that 67.8% of deaths from ill-defined causes in 2020 occurred in those aged 80 years or older, and that senility (one of the specific causes of “ill-defined cause” mortality) accounted for nearly half (49.8%) of these deaths (see Supplementary Tables S7 and S8). Although additional investigations are needed, our results imply the possibility that older individuals at the end of life may have reduced their hospital visits due to the pandemic. Thus, exact causes of death might not be reported accurately for this population. Therefore, we cautiously surmise that these increased cases of ill-defined mortality may have been related to increased deaths due to ill-defined causes during the pandemic.

We also found a disproportionate impact of the pandemic regarding individual characteristics associated with socioeconomic inequality. First, we found that an increase in mortality during the pandemic was evident in those aged 65–79 years, but we did not detect obvious excess mortality in those aged 80 years or older. We conjecture that this may be related to the fact that the young older population (i.e., those less than 80 years of age [approximately]) may be more likely to delay or cancel their medical care services voluntarily or involuntarily. In other words, we conjectured that the results might be related to the fact that most medical services prioritized older populations and COVID-19 patients during the pandemic period. Also, considering the “depletion of susceptible” or “healthy survivor” effect, those in the very old age group may be less susceptible to risk factors that can lead to death than those who died earlier in life [4]; however, more in-depth studies are required in the future to support this conjecture*.*

The above speculation is substantiated by the following figures. In Korea, hospital visits, hospitalisations, and emergency department (ED) visits during the pandemic decreased to a greater degree in those aged 65–79 years than in those aged 80 years or older [33–35], In addition, the impact of restricted medical access on deaths in those aged 65–79 years can be inferred given that the leading causes of death in this age group in 2020 were diseases that require regular and timely care, such as neoplasms and circulatory diseases (see Supplementary Table S7). Moreover, when investigating the older population, the “depletion of susceptibles” or “healthy survivor” effect should be kept in mind; more specifically, survival to very old age may indicate that individuals are less susceptible to risk factors that can lead to death, including the impact of COVID-19 pandemic, than are those who died earlier in life [36, 37].

One of our main findings was that excess deaths due to the pandemic were more prominent in those with low educational levels and in the single population, and that this pattern was common to most causes of death. Previous studies have reported that people with low educational levels (i.e. a proxy for low socioeconomic status) generally have worse health outcomes as well as more limited access to health care resources as compared with highly educated people [38]. Single people are also likely to have worse health status than married people, although there is no consensus as to whether marriage provides a protective effect against adverse health outcomes or whether less healthy or socially disadvantaged individuals are more likely to remain unmarried [39]. It should also be considered that unmarried people may have lived with their parents and received protection from their families [40], but it was not observed in this study. Moreover, during the pandemic, single or unmarried people might become more socially isolated, and people with lower socioeconomic status might face more threats to health, including reduced necessary care, unemployment, financial insecurity, lack of psychosocial resources, and less healthy lifestyles [9].

Regarding the temporal trend in regard to the impact of the pandemic on all-cause mortality, we found that the associated excess risk increased during the 2^nd^ wave of the pandemic period and then sharply decreased during the 3^rd^ wave, resulting in an offset of the total excess in mortality. This reflects the fact that the trend in total deaths during the period corresponding to the 3^rd^ wave (October 26 to December 31) in 2020 was lower than that in the previous period (see Supplementary Fig. S1). In particular, our results imply that the reduction in all-cause mortality in the winter season, corresponding to the 3^rd^ wave in this study, may be associated with a prominent decrease in mortality from respiratory diseases during that period (Supplementary Table S3). Preventive behaviours for ameliorating the spread of the pandemic, such as wearing masks and maintaining personal hygiene, can reduce the risk of infection-related mortality, and these effects might be more pronounced in the winter (when respiratory infections commonly occur) [8]. Nevertheless, this study only investigated trends during the first year of the COVID-19 pandemic, and additional studies are needed to explore long-term trends in excess mortality attributable to the pandemic.

Some limitations of our study must be acknowledged when interpreting the findings reported herein. First, we did not account for seasonal influenza activity and other time-varying confounders, which can affect the relationship between COVID-19 and mortality, as relevant data were not available. Future studies should consider how the time-varying confounders can be controlled in the model. Second, in addition to the insufficient confounders, our study design (an ecological study with a time-series design) is limited in showing the causal effect of COVID-19. Therefore, further studies with more elaborate data and robust methods for counterfactual analyses, such as the synthetic control method. Finally, we only examined excess deaths during the COVID-19 period in 2020, which may be insufficient to capture the prolonged effects of the pandemic on mortality. This issue can be addressed by additional investigations regarding trends in excess mortality due to the pandemic over a longer period.

Despite these drawbacks, a notable strength of our study is the application of a cutting-edge two-stage interrupted time-series design that allows for flexible estimation of excess mortality and adjusts for temporal trends and variations in known risk factors. Another major strength of our study is that we performed this analysis using officially reported nationwide death data with daily count units and stratified the primary findings by cause of death as well as by individual characteristics, thus offering comprehensive and evidence-based information on the impact of the pandemic in Korea in regard to informing future public health research, policy decision-making, and resource allocation.

## Conclusion

In conclusion, our study indicates that no excess in all-cause deaths occurred during the COVID-19 pandemic period in Korea during the year 2020, although differential risks of mortality were evident across specific causes of death and individual characteristics. The findings of our study highlight the need for efforts to address disproportionate access to medical care as well as inequities in health status that have been exacerbated by the pandemic and likewise provide important information regarding the allocation of resources for interventions aimed at addressing inequities in medical and socioeconomic status.

## Supplementary Information

**Additional file 1:** **Supplementary Table S1.** Causes of death and corresponding ICD-10 codes. **Supplementary Table S2.** Characteristics of the 16 regions in Korea. Supplementary Methods. **Supplementary Table S3.** Number of total deaths and estimated excess deaths (with 95% empirical confidence intervals) during the COVID-19 pandemic period (February 18 to December 31, 2020) in Korea by cause of death and phase of the pandemic. **Supplementary Table S4.** Percentage excess in mortality (with 95% empirical confidence interval) during the COVID-19 pandemic period (February 18 to December 31, 2020) in Korea by cause of death and individual characteristic. **Supplementary Table S5. **Percentage excess in mortality (with 95% empirical confidence interval) by cause of death for main model and each sensitivity analysis. **Supplementary Table S6.** Percentage excess in mortality (with 95% empirical confidence interval) by individual characteristic for main model and each sensitivity analysis. **Supplementary Table S7.** Number of total deaths (%) during the COVID-19 pandemic period (February 18 to December 31, 2020) in Korea by cause of death and individual characteristic. **Supplementary Table S8.** Number of total deaths (%) by main specific causes of Symptoms, signs and abnormal clinical and laboratory findings, not elsewhere classified (R00–R99) in 2020. **Supplementary Figure S1.** Temporal trends of total deaths during the study period (2015–2020).

## Data Availability

The data used for this study will be made available to other researchers upon reasonable request. Data for this study has been provided by Statistics Korea, Korea Disease Control and Prevention Agency, and Korea Meteorological Administration, and the data are publicly available.
