# Supplementary material for: Excess mortality and the COVID-19 pandemic: causes of death and social inequalities
Source: BMC Public Health. 2022 Dec 7;22:2293. doi: 10.1186/s12889-022-14785-3 (PMC9730675; doi:10.1186/s12889-022-14785-3)
Supplement: Supplementary file 1 — Additional file 1: Supplementary Table S1. Causes of death and corresponding ICD-10 codes. Supplementary Table S2. Characteristics of the 16 regions in Korea. Supplementary Methods. Supplementary Table S3. Number of total deaths and estimated excess deaths (with 95% empirical confidence intervals) during the COVID-19 pandemic period (February 18 to December 31, 2020) in Korea by cause of death and phase of the pandemic. Supplementary Table S4. Percentage excess in mortality (with 95% empirical confidence interval) during the COVID-19 pandemic period (February 18 to December 31, 2020) in Korea by cause of death and individual characteristic. Supplementary Table S5. Percentage excess in mortality (with 95% empirical confidence interval) by cause of death for main model and each sensitivity analysis. Supplementary Table S6. Percentage excess in mortality (with 95% empirical confidence interval) by individual characteristic for main model and each sensitivity analysis. Supplementary Table S7. Number of total deaths (%) during the COVID-19 pandemic period (February 18 to December 31, 2020) in Korea by cause of death and individual characteristic. Supplementary Table S8. Number of total deaths (%) by main specific causes of Symptoms, signs and abnormal clinical and laboratory findings, not elsewhere classified (R00–R99) in 2020. Supplementary Figure S1. Temporal trends of total deaths during the study period (2015–2020). [file 12889_2022_14785_MOESM1_ESM.docx]

**Excess mortality and COVID-19 pandemic: causes of death and social inequalities**

**Supplementary Materials**

**Table of contents**

Supplementary Table S1. Causes of death and corresponding ICD-10 codes 1

Supplementary Table S2. Characteristics of the 16 regions in Korea. 2

Supplementary Methods 3

Supplementary Table S3. Number of total deaths and estimated excess deaths (with 95% empirical confidence intervals) during the COVID-19 pandemic period (February 18 to December 31, 2020) in Korea by cause of death and phase of the pandemic. 5

Supplementary Table S4. Percentage excess in mortality (with 95% empirical confidence interval) during the COVID-19 pandemic period (February 18 to December 31, 2020) in Korea by cause of death and individual characteristic. 6

Supplementary Table S5. Percentage excess in mortality (with 95% empirical confidence interval) by cause of death for main model and each sensitivity analysis. 7

Supplementary Table S6. Percentage excess in mortality (with 95% empirical confidence interval) by individual characteristic for main model and each sensitivity analysis. 8

Supplementary Table S7. Number of total deaths (%) during the COVID-19 pandemic period (February 18 to December 31, 2020) in Korea by cause of death and individual characteristic. 9

Supplementary Table S8. Number of total deaths (%) by main specific causes of Symptoms, signs and abnormal clinical and laboratory findings, not elsewhere classified (R00–R99) in 2020. 10

Supplementary Figure S1. Temporal trends of total deaths during the study period (2015–2020). 11

Supplementary References 12

**Supplementary Table S1. Causes of death and corresponding ICD-10 codes.**

| **Causes of death** | **International Classification of Disease 10 (ICD-10) codes** |
| --- | --- |
| Certain infectious and parasitic diseases | A00–B99 |
| Neoplasms | C00–D48 |
| Endocrine, nutritional and metabolic diseases | E00–E90 |
| Diseases of circulatory system | I00–I99 |
| Diseases of respiratory system | J00–J99 |
| Diseases of the genitourinary system | N00–N99 |
| Symptoms, signs and abnormal clinical and laboratory findings, not elsewhere classified | R00–R99 |
| Injury, poisoning and certain other consequences of external causes | S00–T98 |
| COVID-19^a^ | U07.1, U07.2 |

^a^Death cases due to COVID-19 were excluded in the analysiss of this study.

**Supplementary Table S2. Characteristics of the 16 regions in Korea.**

| **Region**  **(shi/do)^a^** | **Number of districts**  **(shi/gun/gu)^a^** | **Population density^b^** | **COVID-19 confirmed cases^c^** | **COVID-19 deaths^d^** | **Total deaths^e^** |
| --- | --- | --- | --- | --- | --- |
| Korea | 229 | 516.3 | 117.2 | 1.7 | 286,748 |
| Seoul | 25 | 16083.1 | 195.2 | 1.8 | 42,745 |
| Busan | 16 | 4430.4 | 54.8 | 1.5 | 21,601 |
| Daegu | 8 | 2757.8 | 320.2 | 8.2 | 13,460 |
| Incheon | 10 | 2775.2 | 96.0 | 0.9 | 14,719 |
| Gwangju | 5 | 2907.0 | 74.2 | 0.4 | 7,370 |
| Daejeon | 5 | 2731.4 | 57.3 | 0.5 | 7,136 |
| Ulsan | 5 | 1080.0 | 58.7 | 2.3 | 5,018 |
| Gyunggi | 41 | 1299.7 | 109.1 | 2.0 | 59,041 |
| Gangwon | 18 | 91.5 | 78.3 | 0.8 | 11,457 |
| Chungbuk | 11 | 215.9 | 72.7 | 1.4 | 10,912 |
| Chungnam | 15 | 282.9 | 73.1 | 1.0 | 16,363 |
| Jeonbuk | 14 | 225.2 | 46.1 | 0.6 | 13,854 |
| Jeonnam | 22 | 151.0 | 30.0 | 0.3 | 16,430 |
| Gyungbuk | 23 | 139.9 | 91.0 | 2.3 | 21,420 |
| Gyungnam | 18 | 318.9 | 39.4 | 0.1 | 21,505 |
| Jeju | 2 | 362.5 | 62.0 | 0.0 | 3,717 |

^a^The 16 regions (shi/do) is the first-level administrative region of Korea and the 229 districts (shi/gun/gu) is the second-level administrative region in Korea.

^b^Population density (people per km^2^) in 2020.

^c^The cumulative COVID-19 confirmed cases during 20 January–31 December 2020 per 100,000 population.

^d^The cumulative COVID-19 deaths during 20 January–31 December 2020 per 100,000 population.

^e^Total deaths during 20 January–31 December 2020.

**Supplementary Methods**

**Two-stage analyses:**

We conducted two-stage interrupted time-series analyses to quantify excess the risk of mortality during the COVID-19 pandemic period as compared with the pre-outbreak period in South Korea, following a methodological approach delineated in previous studies^1,2^. In the first stage, a quasi-Poisson regression model was applied to each of the 16 regions in Korea^3^. We included the number of days from the first COVID-19 confirmed case to estimate the time-varying risk during the outbreak period (January 20 to December 31, 2020), using constrained quadratic B-spline function with four equally spaced knots. This function enables the excess risk starting from null at the initial outbreak date and changing flexibly until the end of the study period. We adjusted for time-varying confounding variables, including the long-term trend, seasonality, and weekly variations; the linear term for date, cyclic cubic B-spline function using five equally spaced knots for days of the year, and dummy indicators for day of the week were included. To control for potential differences in underlying mortality caused by non-optimal weather between the pre-outbreak and outbreak periods, we modeled the relationship between average daily temperature and mortality using a cross-basis term of distributed lag non-linear model. The term includes exposure-response association and lag-response association, describing non-linear and delayed effects^4^. The exposure-response association was evaluated using quadratic B-spline with three internal knots set at the 10^th^, 75^th^, and 90^th^ percentiles of region-specific temperature distributions. The lag-response association was calculated using a natural cubic B-spline with an intercept. Three internal knots were equally spaced in the log scale and the lag period was set to 0-21 days^5^.

In the second stage, we pooled the region-specific coefficients of excess risk obtained during the COVID-19 period to the nationwide level, using a mixed-effects multivariate meta-analysis approach^6^. The best linear unbiased prediction (BLUP) was then calculated for each of the 16 regions. This approach can stabilise the variability due to the large differences in population size between regions, leading to more precise estimates^7^.

**The definition of COVID-19 period:**

The COVID-19 outbreak in Korea began on 20 January, when the first confirmed case occurred. In this study, we calculated the number of excess deaths from the epidemic onset date (February 18, 2020), when large-scale outbreaks occurred in Daegu and Gyungbuk, to the end of the study period (December 31, 2020)^8^. Then, we divided the period for quantifying the excess deaths into three waves and two plateaus by defining the wave as a period the number of confirmed cases per moving-averaged seven days was 100 or more and the plateau as a period between waves; the first wave (February 18 to March 17, 2020), the first plateau (March 18 to August 14, 2020), the second wave (August 15 to September 22, 2020), the second plateau (September 23 to October 25, 2020), and the third wave (October 26 to December 31, 2020).

**Supplementary Table S3. Number of total deaths and estimated excess deaths (with 95% empirical confidence intervals) during the COVID-19 pandemic period (February 18 to December 31, 2020) in Korea by cause of death and phase of the pandemic.**

|  | **1^st^ wave** | | **1^st^ plateau** | | **2^nd^ wave** | | **2^nd^ plateau** | | **3^rd^ wave** | |
| --- | --- | --- | --- | --- | --- | --- | --- | --- | --- | --- |
|  | **18 Feb–17 Mar** | | **18 Mar–14 Aug** | | **15 Aug–22 Sep** | | **23 Sep–25 Oct** | | **26 Oct–31 Dec** | |
|  | **Total** | **Excess** | **Total** | **Excess** | **Total** | **Excess** | **Total** | **Excess** | **Total** | **Excess** |
| All causes | 24,607 | 64 (-497 to 551) | 118,871 | -205 (-1,469 to 1,130) | 32,006 | 1,092 (541 to 1,646) | 27,743 | 881 (441 to 1,309) | 57,205 | -1,169 (-2,456 to 139) |
| Certain infectious  and parasitic diseases | 740 | 8 (-75 to 80) | 3,743 | 137 (-340 to 556) | 983 | 23 (-110 to 141) | 882 | 26 (-101 to 137) | 1,784 | 27 (-279 to 316) |
| Neoplasms | 6,647 | 137 (-18 to 279) | 33,888 | 82 (-558 to 723) | 9,084 | 55 (-254 to 392) | 7,583 | -18 (-320 to 293) | 15,366 | -222 (-981 to 520) |
| Endocrine, nutritional  and metabolic diseases | 803 | 31 (-26 to 79) | 3,883 | 362 (185 to 512) | 1,031 | 146 (41 to 239) | 900 | 120 (61 to 172) | 1,960 | 150 (3 to 269) |
| Diseases of  circulatory system | 5,107 | -81 (-313 to 131) | 24,211 | 309 (-400 to 996) | 6,495 | 451 (115 to 721) | 5,673 | 315 (-36 to 607) | 11,839 | -250 (-698 to 156) |
| Diseases of  respiratory system | 3,147 | -285 (-647 to 18) | 13,471 | -2,259 (-3,123 to -1,464) | 3,639 | -204 (-422 to 14) | 3,174 | -144 (-260 to -34) | 6,384 | -1,479 (-1,861 to -1,135) |
| Diseases of  genitourinary system | 746 | 29 (-100 to 145) | 3,532 | -24 (-483 to 372) | 977 | 26 (-108 to 142) | 849 | 43 (-47 to 131) | 1,852 | 6 (-255 to 242) |
| Symptoms, signs and  abnormal clinical and  laboratory findings, not elsewhere classified | 2,483 | 105 (-22 to 218) | 11,942 | 940 (393 to 1,444) | 3,270 | 396 (272 to 486) | 3,019 | 437 (342 to 517) | 6,834 | 878 (629 to 1,124) |
| Injury, poisoning and  certain other consequences of external causes | 2,104 | -27 (-203 to 130) | 10,896 | -191 (-787 to 352) | 2,937 | 45 (-129 to 199) | 2,465 | 14 (-118 to 135) | 4,622 | -264 (-508 to -31) |

**Supplementary Table S4. Percentage excess in mortality (with 95% empirical confidence interval) during the COVID-19 pandemic period (February 18 to December 31, 2020) in Korea by cause of death and individual characteristic.**

|  |  | **Certain infectious and parasitic diseases** | **Neoplasms** | **Endocrine, nutritional**  **and metabolic diseases** | **Diseases of**  **circulatory system** | **Diseases of**  **respiratory system** | **Diseases of**  **genitourinary system** | **Symptoms, signs and abnormal clinical and laboratory findings, not elsewhere classified** | **Injury, poisoning and certain other consequences of external causes** |
| --- | --- | --- | --- | --- | --- | --- | --- | --- | --- |
| Total |  | 2.8 (-8.9 to 16.1) | 0.0 (-2.4 to 2.9) | 10.4 (5.6 to 14.4) | 1.4 (-1.9 to 4.7) | -12.8 (-15.5 to -10.4) | 1.0 (-9.9 to 13.1) | 11.1 (7.9 to 14) | -1.8 (-6.4 to 2.9) |
| Age | <65 | -1.3 (-19.9 to 12.8) | -0.7 (-4.7 to 3.3) | 13.8 (-5.3 to 31) | 2.9 (-2.9 to 7.7) | -7.4 (-16.3 to 0.3) | 16 (-10.8 to 45.7) | -0.4 (-9.2 to 7.1) | -2.7 (-7.3 to 2.0) |
|  | 65–79 | 6.4 (-11.4 to 23.5) | 1.9 (-1.7 to 5.4) | 12.8 (-2.9 to 29.6) | 1.1 (-5.4 to 8.2) | -8.9 (-16.5 to -2.4) | 2.3 (-11.5 to 18.9) | 7.8 (-5.5 to 21.6) | -2 (-6.5 to 2.0) |
|  | ≥80 | 1.9 (-6.4 to 9.1) | -2.6 (-5.1 to -0.2) | 8.3 (-2.6 to 20.6) | 0.8 (-0.9 to 2.2) | -14.8 (-19.9 to -9.7) | -2.2 (-14.1 to 9.9) | 15 (8.4 to 20.8) | 2.4 (-9.1 to 12.2) |
| Education | ≤Elementary school | 4.7 (-7.4 to 17.2) | 1.8 (-0.9 to 4.8) | 11.1 (-1.2 to 24.5) | 1.5 (-3.6 to 6.8) | -11.9 (-16.1 to -7.6) | -0.7 (-7.1 to 3.9) | 14.5 (11.3 to 17.3) | 1.2 (-5.0 to 7.1) |
|  | Middle school | 4 (-18.2 to 27.0) | 3.1 (-0.6 to 6.7) | 7.6 (-15.0 to 30.0) | 3.1 (-7.9 to 14.4) | -11.2 (-18.3 to -5.2) | -0.6 (-13.7 to 10.4) | 9.9 (-2.0 to 21) | 1.2 (-12.9 to 15.8) |
|  | High school | 2 (-4.4 to 6.6) | -1.1 (-3.4 to 1.2) | 15.4 (2.4 to 28.8) | 0.8 (-6.0 to 7.9) | -17.1 (-25.9 to -7.8) | 3.1 (-7.3 to 11.1) | 3 (-7.7 to 14.3) | -3.3 (-9.7 to 2.9) |
|  | ≥College | -5.1 (-15.8 to 2.9) | -5.3 (-8.2 to -2.5) | 15.5 (-6.4 to 38) | -0.3 (-8.8 to 8.2) | -14.5 (-18.8 to -10.9) | 15.6 (-8.9 to 37.9) | 1.1 (-6.2 to 8.1) | -5.7 (-7.7 to -4.7) |
| Marital status | Single | 6.9 (-20.3 to 29.6) | 4.4 (-4.6 to 13.5) | 28.5 (9.6 to 43.8) | 4.3 (-5.9 to 14.9) | -6.1 (-21.1 to 8.3) | 27.2 (4.7 to 39.2) | 6.6 (-2.7 to 14.0) | 3.7 (0.3 to 6.9) |
|  | Married | 2.8 (-11.5 to 17.5) | 0.9 (-2.5 to 4.2) | 10.4 (0.7 to 20.6) | 2.6 (-1.0 to 6.3) | -11.9 (-17.3 to -6.9) | 5.3 (-7.0 to 16.6) | 11.8 (-0.1 to 23.9) | -5 (-10.9 to 0.4) |
|  | Others (divorced/widowed) | 3.5 (-10.5 to 20.0) | -2.1 (-4.8 to 0.3) | 9.6 (2.4 to 16.4) | 0.3 (-6.0 to 6.6) | -13.7 (-20 to -7.3) | -2.4 (-13.6 to 9.2) | 11.6 (4.5 to 19.1) | -0.7 (-3.6 to 1.6) |

**Supplementary Table S5. Percentage excess in mortality (with 95% empirical confidence interval) by cause of death for main model and each sensitivity analysis.**

|  | **Main** | **Sensitivity analysis** | | | | | |
| --- | --- | --- | --- | --- | --- | --- | --- |
|  |  | **The number of knots in the quadratic B-spline function for days from the first COVID-19 confirmed case** | | **The number of knots in cyclic B-spline function for days of the year** | | **The days of lag period in distributed lag non-linear model** | |
|  |  | **5** | **6** | **4** | **6** | **14** | **28** |
| Total causes | 0.3 (-0.9 to 1.4) | 0.3 (-0.8 to 1.3) | 0.3 (-0.8 to 1.4) | 0.2 (-0.9 to 1.5) | 0.2 (-0.8 to 1.2) | -0.1 (-0.8 to 0.6) | 0.7 (-0.3 to 1.8) |
| Certain infectious and parasitic diseases | 2.8 (-8.9 to 16.1) | 2.8 (-0.5 to 5) | 2.9 (-8.6 to 14.5) | 2.8 (-6.2 to 10.8) | 2.6 (-8.9 to 15.1) | 2.3 (-2.6 to 6) | 3.4 (-5.9 to 13.8) |
| Neoplasms | 0.0 (-2.4 to 2.9) | 0.1 (-2.6 to 2.4) | 0.1 (-1.9 to 1.9) | 0 (-2.3 to 2.2) | 0 (-2.1 to 2.3) | -0.3 (-2.4 to 1.8) | 0.1 (-2.3 to 2.7) |
| Endocrine, nutritional and metabolic diseases | 10.4 (5.6 to 14.4) | 10.5 (3 to 18.2) | 10.4 (5.5 to 13.9) | 10.4 (0.6 to 21) | 10.3 (4.1 to 15.1) | 9.8 (3.4 to 15) | 11.7 (3.6 to 19.8) |
| Diseases of circulatory system | 1.4 (-1.9 to 4.7) | 1.5 (-1.8 to 4.6) | 1.4 (-1 to 3.6) | 1.4 (-2.7 to 5.7) | 1.3 (-2.9 to 5.8) | 1.2 (-1.1 to 3.2) | 1.9 (-0.3 to 4) |
| Diseases of respiratory system | -12.8 (-15.5 to -10.4) | -12.8 (-14.8 to -10.9) | -12.8 (-14.4 to -11.4) | -12.9 (-16.2 to -9.9) | -13 (-16.6 to -9.5) | -13.6 (-16.1 to -11.6) | -11.8 (-14.1 to -9.6) |
| Diseases of the genitourinary system | 1.0 (-9.9 to 13.1) | 1.1 (-2.7 to 4.3) | 1.2 (-7.2 to 10.4) | 1 (-2.9 to 3.9) | 1 (-9.5 to 12.6) | 1 (-7.6 to 10.2) | 1.3 (-5.8 to 7.7) |
| Symptoms, signs and abnormal clinical and laboratory findings, not elsewhere classified | 11.1 (7.9 to 14) | 11.1 (3.1 to 20.3) | 11.1 (6.5 to 15.3) | 10.9 (3.6 to 19.2) | 11.1 (8.2 to 13.6) | 10.6 (4.6 to 16.9) | 11.4 (5.1 to 17.3) |
| Injury, poisoning and certain other consequences of external causes | -1.8 (-6.4 to 2.9) | -1.8 (-6.4 to 2.6) | -1.8 (-7.8 to 4.1) | -1.9 (-5.8 to 2) | -1.8 (-5.1 to 1.1) | -1.8 (-5 to 0.9) | -1.5 (-5.1 to 1.6) |

**Supplementary Table S6. Percentage excess in mortality (with 95% empirical confidence interval) by individual characteristic for main model and each sensitivity analysis.**

|  |  | **Main** | **Sensitivity analysis** | | | | | |
| --- | --- | --- | --- | --- | --- | --- | --- | --- |
|  |  |  | **The number of knots in the quadratic B-spline function for days from the first COVID-19 confirmed case** | | **The number of knots in cyclic B-spline function for days of the year** | | **The days of lag period in distributed lag non-linear model** | |
|  |  |  | **5** | **6** | **4** | **6** | **14** | **28** |
| Total |  | 0.3 (-0.9 to 1.4) | 0.3 (-0.8 to 1.3) | 0.3 (-0.8 to 1.4) | 0.2 (-0.9 to 1.5) | 0.2 (-0.8 to 1.2) | -0.1 (-0.8 to 0.6) | 0.7 (-0.3 to 1.8) |
| Sex | Males | 0.4 (-0.7 to 1.4) | 0.4 (-0.8 to 1.7) | 0.4 (-0.8 to 1.7) | 0.4 (-0.9 to 1.6) | 0.3 (-0.3 to 0.9) | 0 (-1.2 to 1.3) | 0.8 (0 to 1.6) |
|  | Females | 0.1 (-1.6 to 1.8) | 0.2 (-1.6 to 2) | 0.2 (-1.9 to 2.1) | 0.1 (-1 to 1) | 0.1 (-1.4 to 1.7) | -0.2 (-1.9 to 1.4) | 0.6 (-1 to 2.3) |
| Age | <65 | -0.4 (-2 to 1.1) | -0.4 (-2 to 1.2) | -0.4 (-1.5 to 0.6) | -0.4 (-2 to 1.1) | -0.4 (-2 to 1.1) | -0.8 (-2.1 to 0.7) | -0.1 (-1.9 to 1.7) |
|  | 65–79 | 1.2 (0.1 to 2.4) | 1.3 (0.2 to 2.3) | 1.3 (0 to 2.5) | 1.2 (0.5 to 1.9) | 1.2 (0.1 to 2.2) | 0.9 (-0.1 to 1.9) | 1.7 (0.6 to 2.8) |
|  | ≥80 | -0.6 (-2.2 to 1.2) | -0.5 (-2.6 to 1.3) | -0.6 (-2.2 to 1.2) | -0.7 (-2.4 to 1.1) | -0.7 (-2.2 to 0.8) | -1 (-2.1 to 0.1) | -0.1 (-2.2 to 1.9) |
| Education | ≤Elementary school | 1.5 (0.3 to 2.6) | 1.5 (-0.6 to 3.6) | 1.5 (-0.6 to 3.6) | 1.4 (-0.6 to 3.5) | 1.4 (0.8 to 1.9) | 1.2 (0.2 to 2) | 2 (1.3 to 2.6) |
|  | Middle school | 1.2 (-1.6 to 3.9) | 1.2 (-0.1 to 2.4) | 1.2 (-1.7 to 4.5) | 1.1 (-1.8 to 4) | 1.2 (-1.9 to 4.3) | 0.9 (-2.5 to 4.1) | 1.6 (-1.7 to 4.8) |
|  | High school | -1.5 (-3.1 to 0.3) | -1.4 (-3.1 to 0.2) | -1.4 (-3.5 to 0.7) | -1.5 (-3.3 to 0.4) | -1.6 (-3.8 to 0.6) | -1.9 (-3.4 to -0.3) | -1 (-2.7 to 0.6) |
|  | ≥College | -4.1 (-6.4 to -1.7) | -4.1 (-6.6 to -1.6) | -4.1 (-5.8 to -2.4) | -4.2 (-6.4 to -2) | -4.1 (-6.5 to -1.4) | -4.6 (-6.9 to -2.4) | -3.9 (-6.1 to -1.6) |
| Marital status | Single | 3.9 (1.9 to 5.9) | 3.9 (2.6 to 5.1) | 3.9 (1.9 to 5.6) | 3.8 (1.7 to 6) | 3.9 (2.1 to 5.5) | 3.6 (1.5 to 5.9) | 4.5 (2.4 to 6.5) |
|  | Married | 0.3 (-0.6 to 1.1) | 0.3 (-0.2 to 0.9) | 0.3 (-0.2 to 0.8) | 0.3 (-0.5 to 1.1) | 0.2 (-0.7 to 1.1) | 0 (-1.1 to 1) | 0.7 (-0.2 to 1.6) |
|  | Others (divorced/widowed) | -0.3 (-2.2 to 1.4) | -0.3 (-1.5 to 0.9) | -0.3 (-2.1 to 1.5) | -0.4 (-1.4 to 0.6) | -0.4 (-1.9 to 1.1) | -0.8 (-1.6 to 0) | 0.1 (-1.6 to 1.7) |

**Supplementary Table S7. Number of total deaths (%) during the COVID-19 pandemic period (February 18 to December 31, 2020) in Korea by cause of death and individual characteristic.**

|  |  | **Certain infectious and parasitic diseases** | **Neoplasms** | **Endocrine, nutritional**  **and metabolic diseases** | **Diseases of**  **circulatory system** | **Diseases of**  **respiratory system** | **Diseases of**  **genitourinary system** | **Symptoms, signs and abnormal clinical and laboratory findings, not elsewhere classified** | **Injury, poisoning and certain other consequences of external causes** |
| --- | --- | --- | --- | --- | --- | --- | --- | --- | --- |
| Total |  | 8,132 (100.0) | 72,568 (100.0) | 8,577 (100.0) | 53,325 (100.0) | 29,815 (100.0) | 7,956 (100.0) | 27,548 (100.0) | 23,024 (100.0) |
| Sex | Males | 3,767 (46.3) | 44,804 (61.7) | 4,355 (50.8) | 25,438 (47.7) | 17,093 (57.3) | 3,499 (44.0) | 12,318 (44.7) | 15,711 (68.2) |
|  | Females | 4,365 (53.7) | 27,764 (38.3) | 4,222 (49.2) | 27,887 (52.3) | 12,722 (42.7) | 4,457 (56.0) | 15,230 (55.3) | 7,313 (31.8) |
| Age | <65 | 1,062 (13.1) | 19,747 (27.2) | 1,567 (18.3) | 8,161 (15.3) | 1,872 (6.3) | 828 (10.4) | 4,061 (14.7) | 13,232 (57.5) |
|  | 65–79 | 2,284 (28.1) | 29,655 (40.9) | 2,785 (32.5) | 14,405 (27.0) | 8,029 (26.9) | 2,274 (28.6) | 4,806 (17.4) | 5,222 (22.7) |
|  | ≥80 | 4,786 (58.9) | 23,166 (31.9) | 4,225 (49.3) | 30,759 (57.7) | 19,914 (66.8) | 4,854 (61.0) | 18,681 (67.8) | 4,570 (19.8) |
| Education | ≤Elementary school | 993 (12.21) | 10,812 (14.9) | 1,194 (13.9) | 6,566 (12.3) | 3,831 (12.9) | 983 (12.4) | 3,018 (11.0) | 3,008 (13.1) |
|  | Middle school | 4,497 (55.3) | 27,110 (37.4) | 4,172 (48.6) | 28,029 (52.6) | 16,555 (55.5) | 4,290 (53.9) | 15,662 (56.9) | 6,336 (27.5) |
|  | High school | 1,390 (17.1) | 18,753 (25.8) | 1,816 (21.2) | 9,921 (18.6) | 4,821 (16.2) | 1,386 (17.4) | 4,459 (16.2) | 7,236 (31.4) |
|  | ≥College | 721 (8.9) | 12,119 (16.7) | 924 (10.8) | 5,847 (11.0) | 3,023 (10.1) | 821 (10.3) | 2,612 (9.5) | 4,947 (21.5) |
|  | Unknown | 531 (6.5) | 3,774 (5.2) | 471 (5.5) | 2,962 (5.6) | 1,585 (5.3) | 476 (6.0) | 1,797 (6.5) | 1,497 (6.5) |
| Marital status | Single | 519 (6.4) | 4,071 (5.6) | 662 (7.7) | 3,054 (5.7) | 1,192 (4.0) | 369 (4.6) | 2,155 (7.8) | 5,713 (24.8) |
|  | Married | 3,216 (39.5) | 43,193 (59.5) | 3,455 (40.3) | 21,739 (40.8) | 13,419 (45) | 3,204 (40.3) | 9,173 (33.3) | 10,180 (44.2) |
|  | Others (divorced/widowed) | 4,373 (53.8) | 25,216 (34.7) | 4,440 (51.8) | 28,432 (53.3) | 15,167 (50.9) | 4,374 (55.0) | 16,148 (58.6) | 7,096 (30.8) |
|  | Unknown | 24 (0.3) | 88 (0.1) | 20 (0.2) | 100 (0.2) | 37 (0.1) | 9 (0.1) | 72 (0.3) | 35 (0.2) |

**Supplementary Table S8. Number of total deaths (%) by main specific causes of Symptoms, signs and abnormal clinical and laboratory findings, not elsewhere classified (R00–R99) in 2020.**

| **Main specific causes** | **Total deaths** |
| --- | --- |
| Total of specific causes | 31,801 (100.00) |
| Symptoms and signs involving the circulatory and respiratory systems |  |
| Other symptoms and signs involving the circulatory and respiratory systems | 1,052 (3.3) |
| General symptoms and signs |  |
| Senility | 15,823 (49.8) |
| Other general symptoms and signs | 2,585 (8.1) |
| Ill-defined and unknown causes of mortality |  |
| Other sudden death, cause unknown | 890 (2.8) |
| Unattended death | 1,293 (4.1) |
| Other ill-defined and unspecified causes of mortality | 9,143 (28.8) |

**Supplementary Figure S1. Temporal trends of total deaths during the study period (2015–2020).**


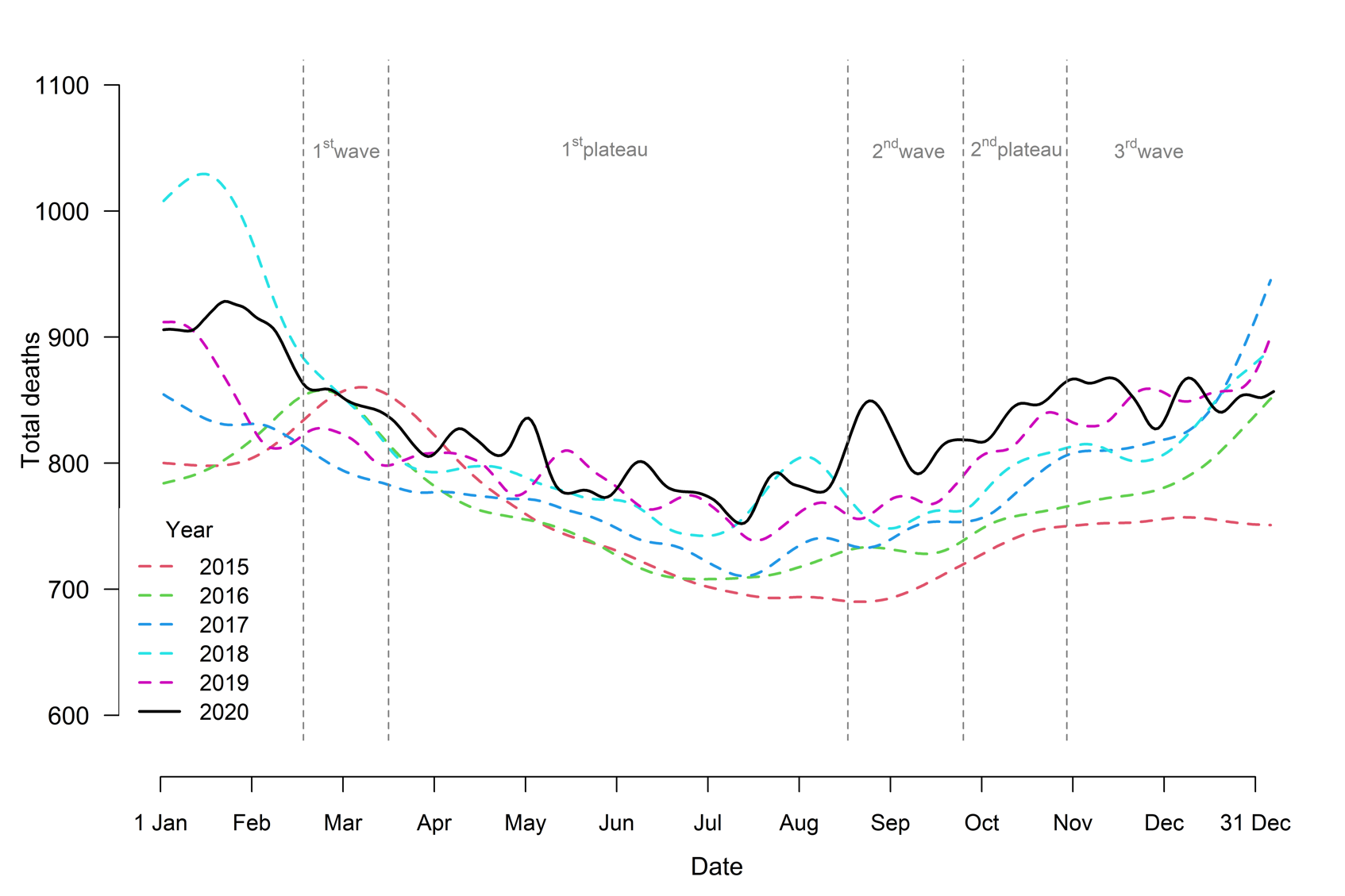


**Supplementary References**

1 Scortichini, M. *et al.* Excess mortality during the COVID-19 outbreak in Italy: a two-stage interrupted time-series analysis. *International journal of epidemiology* **49**, 1909-1917 (2020).

2 Onozuka, D. *et al.* Reduced mortality during the COVID-19 outbreak in Japan, 2020: a two-stage interrupted time-series design. *International journal of epidemiology* **51**, 75-84 (2022).

3 Bhaskaran, K., Gasparrini, A., Hajat, S., Smeeth, L. & Armstrong, B. Time series regression studies in environmental epidemiology. *International journal of epidemiology* **42**, 1187-1195 (2013).

4 Gasparrini, A. *et al.* Mortality risk attributable to high and low ambient temperature: a multicountry observational study. *The lancet* **386**, 369-375 (2015).

5 Gasparrini, A. Modeling exposure–lag–response associations with distributed lag non‐linear models. *Statistics in medicine* **33**, 881-899 (2014).

6 Sera, F., Armstrong, B., Blangiardo, M. & Gasparrini, A. An extended mixed‐effects framework for meta‐analysis. *Statistics in Medicine* **38**, 5429-5444 (2019).

7 Gasparrini, A., Armstrong, B. & Kenward, M. G. Multivariate meta‐analysis for non‐linear and other multi‐parameter associations. *Statistics in medicine* **31**, 3821-3839 (2012).

8 Kim, Y. *et al.* COVID-19 1-year outbreak report as of January 19, 2021 in the Republic of Korea. *Public Health Wkly Rep* **14**, 472-481 (2021).
